# Biological characteristics of the bacteriophage LDT325 and its potential application against the plant pathogen *Pseudomonas syringae*

**DOI:** 10.3389/fmicb.2024.1370332

**Published:** 2024-03-12

**Authors:** Li Liu, Bing Wang, Anqi Huang, Hua Zhang, Yubao Li, Lei Wang

**Affiliations:** College of Agriculture and Agricultural Engineering, Liaocheng University, Liaocheng, China

**Keywords:** *Pseudomonas syringae*, bacteriophage, biocontrol, tea bud blight, bacterial disease

## Abstract

Bud blight disease caused by *Pseudomonas syringae* is a major bacterial disease of tea plants in China. Concerns regarding the emergence of bacterial resistance to conventional copper controls have indicated the need to devise new methods of disease biocontrol. Phage-based biocontrol may be a sustainable approach to combat bacterial pathogens. In this study, a *P. syringae* phage was isolated from soil samples. Based on morphological characteristics, bacteriophage vB_PsS_LDT325 belongs to the Siphoviridae family; it has an icosahedral head with a diameter of 53 ± 1 nm and nonretractable tails measuring 110 ± 1 nm. The latent period and burst size of the phage were 10 min and 17 plaque-forming units (PFU)/cell, respectively. Furthermore, an analysis of the biological traits showed that the optimal multiplicity of infection (MOI) of the phage was 0.01. When the temperature exceeded 60°C, the phage titer began to decrease. The phage exhibited tolerance to a wide range of pH (3–11) and maintained relatively stable pH tolerance. It showed a high tolerance to chloroform, but was sensitive to ultraviolet (UV) light. The effects of phage LDT325 in treating *P. syringae* infections *in vivo* were evaluated using a tea plant. Plants were inoculated with 2 × 10^7^ colony-forming units (CFU)/mL *P. syringae* using the needle-prick method and air-dried. Subsequently, plants were inoculated with 2 × 10^7^ PFU/mL LDT325 phage. Compared with control plants, the bacterial count was reduced by 1 log10/0.5 g after 4 days in potted tea plants inoculated with the phage. These results underscore the phage as a potential antibacterial agent for controlling *P. syringae*.

## 1 Introduction

The tea plant, belonging to the tea family, is an upright evergreen plant with simple alternate leaves. Globally, the tea plant is extensively found in tropical and subtropical regions. Tea foliage is considered to possess medicinal significance. Tea contains robust compounds such as carotenoids, catechins, and lignans, which potentially confer defense against cancer (Hastak et al., 1997; Abdullaev, 2002). Because tea plants primarily grow in subtropical and tropical regions, a warm and humid climate is conducive to the proliferation and spread of pathogens, which cause various diseases in tea plants. Recently, an increase in the prevalence of bacterial bud blight in tea plants has been noted in China. The disease is caused by the bacteria *P. syringae*, which was isolated in New Zealand by Hale (1975), who described the pathogen. Subsequently, the bacterium was classified as a new pathogen, *P. syringae*. Currently, diseases caused by *P. syringae* have spread worldwide, including the United States (Koike et al., 1999), Australia (Noble et al., 2006), and Korea (Myung et al., 2011). Typically, diseases affecting the leaves directly impact the tea yield, whereas those affecting the stalk and roots impact the overall survival of tea plants (Balestra et al., 2009; Ferrante and Scortichini, 2009). Tea bud blight mainly affects the tender buds and leaves. After the bud tip is damaged, the leaves turn dark brown, shrink, and are unable to stretch, and the whole tender tip is dead in severe cases. Tea plantations afflicted with bud blight may experience reductions in productivity and quality (Khandan et al., 2013; Bartoli et al., 2015).

*P. syringae*, a widespread gram-negative bacterium can persist in the environment or remain dormant in plant tissues during winter. It can spread through wind or rain. Environmental conditions that affect bacterial colonization and disease occurrence require a combination of moderate temperature and moisture on leaves. The appearance and expansion of *P. syringae* in all major tea producing regions worldwide have negatively impacted the sustainability of tea plants (Balestra et al., 2009; Ferrante and Scortichini, 2009). *P. syringae* is extremely aggressive, spreading rapidly among plants of diverse varieties (Khandan et al., 2013; Bartoli et al., 2015). Antibiotics and copper-based substances are the primary antimicrobials used for disease biocontrol. However, their usage can harm beneficial microbial communities and promote the development of resistant strains (Lee et al., 1994). Conventional approaches for the biocontrol of bacterial bud blight in tea plants, such as using copper-based bactericides, are becoming less desirable because of the development of copper-resistant bacteria and the environmental harm caused by copper accumulation (Sundin and Bender, 1993; Jones et al., 2007). Consequently, their use is limited in Europe. Although using chemical pesticides has helped mitigate the harm caused by plant diseases, environmental pollution and threats to human health due to pesticide residues have reached alarming levels.

One potential substitute for managing bacterial diseases is the use of bacteriophages. Phages are among the most abundant and diverse microorganisms in the environment. They are viruses specifically target and infect bacteria, without harming plants. Phages targeting pathovars of *P. syringae* have been investigated as potential biocontrol agents (Di Lallo et al., 2014; Doss et al., 2017). Because of their high host specificity and capacity to multiply, bacteriophages are considered potential candidates for treating phytopathogenic bacterial diseases (Buttimer et al., 2017).

This study aimed to isolate and characterize phages infectious to *P. syringae* as potential biocontrol agents. One strain of *P. syringae*-targeting phage was isolated and purified from the soil at the flower base. The biological characteristics of phage LDT325, including morphology, titer, one-step growth curve, thermal and pH stability were determined. Chloroform and UV stability were also determined. More importantly, *in vitro* and *in vivo* experiments on phage effectiveness against *P. syringae* were determined. Results showed that the phage could reduce the number of *P. syringae* in tea plants. Therefore, phage LDT325 can be a potential biocontrol agent against *P. syringae* in tea plants.

## 2 Materials and methods

### 2.1 Microbial strains, bacteriophage strain, and cultivation conditions

The microbial strain used in this study was isolated from diseased spots on tea leaves. Isolated bacteria were identified as *P. syringae* via molecular identification and routinely cultured in Luria–Bertani (LB) liquid medium at 30°C. The *P. syringae* phage LDT325 was isolated from soil sampled from the flower base in Dongchangfu District, Liaocheng City, Shandong Province, China. The tested tea variety was Longjing No. 43 (*Camellia sinensis*), purchased from Juchuang Agricultural Development Co., Ltd., Shaoxing City, Zhejiang Province, China.

### 2.2 Isolation and purification of bacteriophage LDT325

Bacteriophages were isolated from the soil of the Liaocheng flower base in Shandong Province. Phage LDT325 was isolated and purified using *P. syringae* as the host. Initially, 5 g of soil was mixed with 5 mL of sterile water and vortexed to achieve a homogeneous mixture. The soil sample was centrifuged at 10,000 × *g* for 5 min. The supernatant was removed and passed through a microporous filter with a pore diameter of 0.22 μm to remove bacteria. The 15 mL filtrate was mixed with 3 mL of exponentially growing bacteria (2 × 10^7^ CFU/mL). The mixture was inoculated into 15 mL of LB liquid medium and then incubated at 30°C for 4 h, centrifuged at 10,000 × *g* for 5 min (centrifuge H1650, Xiangyi, China), and filtered through a 0.22-μm filter to obtain the supernatant. Phage separation was conducted using the double-layer agar (DLA) plate method. Then, 200 μL of log-phase bacteria (2 × 10^7^ CFU/mL) were incubated with 5 mL of soft agar [LB containing 0.4% (w/v) agar] and poured onto solid bottom agar [LB containing 1.5% (w/v) agar]. Approximately 5 μL of the filtration solution was applied on to the solidified LB agar plate and incubated for 12 h at 30°C. Agar petri dishes were assessed for the presence of see-through regions or formations at the inoculation points.

The purified bacteriophage was obtained using the dual-layer agar plate method. See-through plaques were collected and resuspended in a test tube containing 1.5 mL of SM buffer. After amplification of the bacteriophage at approximately 25°C, the obtained solution was centrifuged for 1 min at 12,000 × *g*. The supernatant was filtered through a 0.22-μm filter. The double-layer plate method was used to titrate the filtered supernatant again. Based on their size and transparency, different plaques were selected after incubation at 30°C for 12 h and were resuspended in 900 μL of SM buffer. The purification process was repeated five times until homogeneously distributed plaque-containing phage isolates were obtained. Phage particles were stored in precooled LB medium, mixed with 50% (v/v) glycerol, and refrigerated at −80°C (Xing et al., 2017; Chen et al., 2018).

### 2.3 Phage infectivity efficiency

Bacteriophage isolation was initiated by incubation with 5 mL of LB broth at 30°C, followed by the collection of the clarified supernatant. The plaque number was counted using the dual-layer agar plate method. The initial bacteriophage titration was computed using the following equation: initial bacteriophage titration = number of plaques × 5× dilution factor.

### 2.4 Transmission electron microscopy

A high-titer phage lysis buffer was prepared via plate amplification. The isolated bacteriophage (2 × 10^7^ PFU/mL) was added to the bacterial solution (2 × 10^7^ CFU/mL) and cultured for 4 h. The solution was mixed with 5 mL of dissolved liquid LB agar [LB containing 0.4% (w/v) agar] and the mixture was poured onto a plate containing LB agar [LB containing 1.5% (w/v) agar]. The plate was incubated at 30°C for 12 h, and 10 mL of SM buffer was added to the culture plate and gently agitated for 4 h. Subsequently, the culture mixture was centrifuged at 10,000 × *g* for 4 min, and the resulting supernatant was filtered through a 0.22-μm membrane. The concentration of the bacteriophage liquid filtrate was determined using the double-agar plate method. A bacteriophage suspension was negatively stained with 2% phosphotungstic acid and viewed using electron microscopy (EM). Images were obtained at an acceleration voltage of 100 kV, and the phage head and tail dimensions were determined using ImageJ (Peng and Yuan, 2018).

### 2.5 Determining optimal multiplicity of infection (MOI)

To determine the optimal MOI, the *P. syringae* bacterial solution was diluted to 2 × 10^7^ CFU/mL. The bacteriophage suspension was mixed with the diluted *P. syringae* solution at ratios of 10, 1, 0.1, and 0.01. The mixtures were incubated for 3 h at 30°C under constant agitation at 180 rpm, centrifuged at 10,000 × *g* for 10 min; and filtered through a 0.22-μm filter. The concentration of the bacteriophage suspension was assessed using the dual-layer agar plate method. The experiment was performed in triplicate. The dilution with the highest number of phage particles was selected as the dilution with the most favorable MOI (Sui et al., 2021).

### 2.6 One-step growth curve

The growth curve was determined as described previously (Cao et al., 2022). Phage LDT325 was mixed with the bacterial solution during the logarithmic growth phase at an MOI of 0.01 (Sun et al., 2012). The mixture was incubated at 37°C for 5 min and centrifuged at 10,000 × *g* for 10 min. The supernatant was removed. The pellet was rinsed twice with LB broth, suspended in an equivalent amount of LB liquid medium, and incubated at 180 rpm under continuous agitation at 30°C. Sampling was performed at 0, 10, 20, 30, 40, 50, 60, 70, 80, 90, 100, 110, 120, 140, 160, 180, and 200 min, and bacteriophage concentrations were determined using the dual-layer agar plate method. After calculating the number of plaque-forming units per milliliter (PFU/mL), the latent period and burst size were determined by dividing the mean PFU/mL during the latent period by that during the last three time points of the experiment (Sanmukh et al., 2023). The experiment was performed in triplicate.

### 2.7 Assessment of thermal and pH stability

Thermal stability was evaluated by incubating purified bacteriophages at different temperatures (40°C, 50°C, 60°C, 70°C, and 80°C). Bacteriophage concentration was measured using the dual-layer agar plate method at 20, 40, and 60 min after incubation (Tie et al., 2018; Shang et al., 2021; Sui et al., 2021). To assess pH sensitivity, purified phages (2 × 10^7^ PFU/mL) were exposed to SM buffer at pH (1–13) for 1 h at 37°C (Tie et al., 2018; Yang et al., 2020; Sui et al., 2021).

### 2.8 Impact of chloroform on phage viability

The phages (2 × 10^7^ PFU/mL) were incubated with 20% chloroform on a shaker for 1 h. The control group lacked chloroform. Bacteriophage concentrations were evaluated using the dual-layer agar plate method and incubated at 30°C for 12 h in triplicate. The experiment was performed three times (Gašić et al., 2022).

### 2.9 Impact of ultraviolet irradiation on phage survival

Further refinement was performed based on the method (Iriarte et al., 2007; Czajkowski et al., 2014) and the impact of ultraviolet (UV) radiation on phage strains was investigated. A UV lamp integrated within a laminar flow hood was used as the UV light source (365 nm, 75 μW/cm^2^). Phage suspensions were prepared at a density of 2 × 10^7^ PFU/mL. In total, 5 mL of phage particles were introduced into each petri dish. The suspensions were irradiated with UV light at a distance of 30 cm for 0, 1, 2, 3, 6, 9, 12, 15, 18, and 21 min. The samples were diluted serially. Subsequently, phage concentrations were determined using the dual-layer agar plate method, and the samples were incubated at 30°C for 12 h. Three replicates of the experiment were performed.

### 2.10 *In vitro* phage effectiveness

The ability of the LDT325 bacteriophage (2 × 10^7^ PFU/mL) to lyse target bacteria was assessed by introducing a bacteriophage (200 μL) sample into a suspension of *P. syringae* (200 μL). An equal volume of LB medium was added to a bacterial culture with no phage inoculum as a control. The mixture was cultured at 30°C under shaking conditions at 180 rpm. The phage bacteriolytic activity was assessed by monitoring the absorbance of the culture solution (OD600) at 6-h intervals for up to 48 h. The assay was performed in triplicate (Wang et al., 2016).

### 2.11 *In vivo* phage efficacy

The susceptible tea cultivar Longjing No. 43 was used as the study material. Tea leaves were sterilized with 75% alcohol, washed multiple times with sterile water, and left to dry. The number of bacteria in leaf tissue was evaluated using the needle-prick method (Aftab et al., 2022). First, 40 μL of *P. syringae* solution (2 × 10^7^ CFU/mL) was applied to the top surface of each tea leaf (dispersed across the four needle puncture apertures) and air-dried. Then, 40 μL of phage suspension (2 × 10^7^ PFU/mL) was applied to the top surface of each tea leaf (dispersed across the four needle puncture apertures), and injured leaves were protected with sterile cotton. Untreated leaves were simultaneously infected with *P. syringae*. The treated leaves were stored in a biochemical incubator at 28°C for 4 days under a relative humidity (RH) of 90%. Leaf samples from each group were collected after infection. For each specimen, 0.5 g of leaf tissue was mixed with 1 mL sterile water to determine the *P. syringae* concentration (CFU/mL). The experiments were performed in triplicate.

### 2.12 Determination of lesion length on tea leaves

The lesion length was determined as described previously (Wang et al., 2017). Before inoculation, the leaves were treated with 75% alcohol, washed multiple times with sterile water, and air-dried. The leaves were punctured four times using a 0.45-mm diameter needle. Then, 40 μL of *P. syringae* solution (2 × 10^7^ CFU/mL) was applied onto the upper surface of each leaf, targeting the four puncture wounds, and the leaf was air-dried. Subsequently, 40 μL of the phage suspension (2 × 10^7^ PFU/mL) was applied onto the upper surface of each tea leaf (targeting the four puncture wounds). The wounded leaves were protected with sterilized cotton, control leaves were inoculated with *P. syringae*, and treated leaves were stored in a biochemical incubator at 28°C for 72 h with a relative humidity of 90%. The size of the disease spot was assessed 6 h after inoculation. The experiment was performed in triplicate.

### 2.13 Hematoxylin and eosin staining

The leaves were fixed with glutaraldehyde for at least 24 h for tissue observation. The fixed tissues were first dehydrated in 75% ethanol for 2 h, followed by 85% ethanol for 2 h, 90% ethanol for 2 h, 95% ethanol for 1 h, and finally anhydrous ethanol for 2 h. The tissues were dehydrated twice with xylene for 45 min and infiltrated with paraffin. Paraffin-embedded tissues were sliced into 4-μm sections using a tissue slicer. The slices were baked at 65°C; deparaffinized twice with xylene; washed twice with anhydrous ethanol for 10 min per rinse; washed with 95, 90, 80, and 70% ethyl alcohol for 5 min; and stained with hematoxylin for 10 min and eosin for 1.5 min. The slices were desiccated via immersion twice in 95% ethanol for 5 min, twice in pure ethanol for 5 min, and twice in xylene for 5 min. The slices were air-dried and sealed with neutral balsam (Liu et al., 2020). Under a Nikon 80i microscope (Japan), the paraffin sections were observed.

### 2.14 Statistical analysis

Data were analyzed using GraphPad Prism 8.0.2, specifically employing one-way analysis of variance. At least three independent replicates were performed under identical conditions, and data were presented as the mean ± standard deviation. Statistical significance was assessed based on *p*-value, and *p*-values of <0.05 were considered to indicate statistical significance. The significance levels were denoted as follows: *p < 0.05, **p < 0.01, ***p < 0.001, and ****p < 0.0001.

## 3 Results

### 3.1 Isolation and purification of *Pseudomonas syringae* phage LDT325

**Figure 1:**
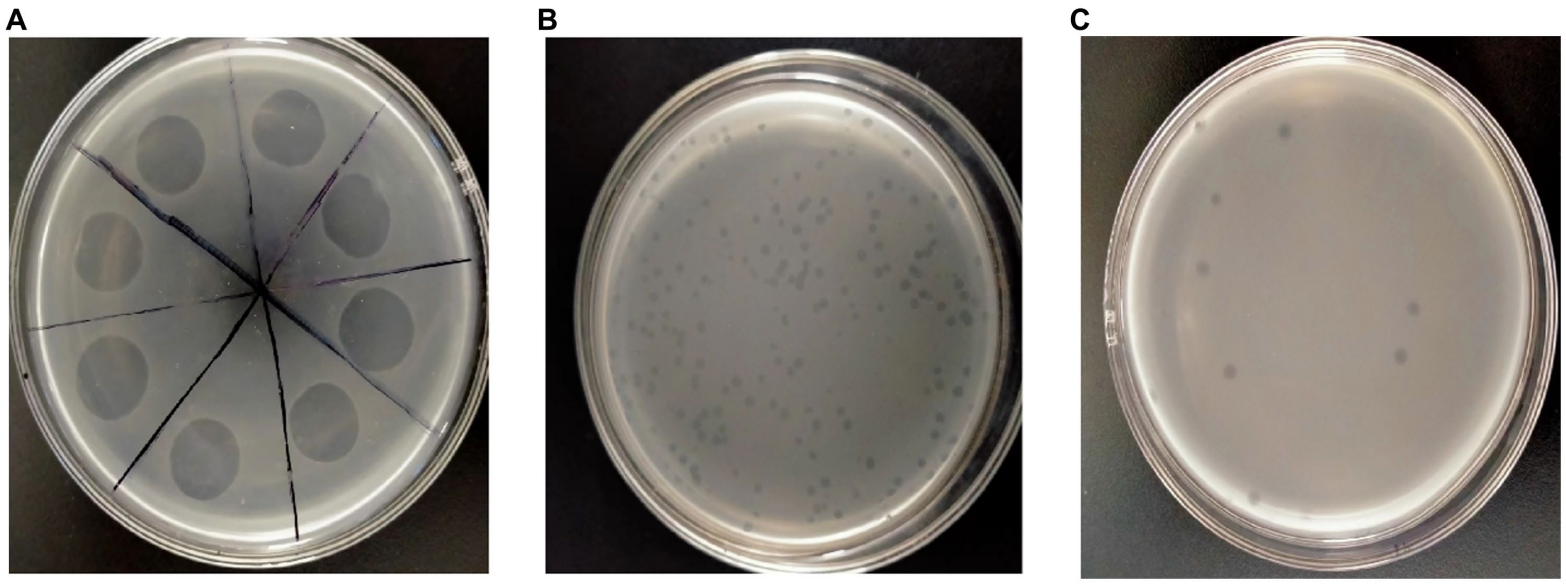
Isolation and purification of the *Pseudomonas syringae* phage vB_PsS_LDT325. **(A)** Sample filtrate displays the clear lysis area on the double-layer plate overlays with suspensions of *Pseudomonas syringae* after incubation for 12 h, depicting lysed bacteria. **(B)** The phage vB_PsS_LDT325 crude filtrate. After 12 h of incubation, clear and different sized plaques were displayed on the *Pseudomonas syringae* lawn. **(C)** The purified phage vB_PsS_LDT325 displays clear and uniform patched plaques on a *Pseudomonas syringae* lawn after incubation for 12 h.

In this study, 12 soil samples were obtained from the Liaocheng flower base in Liaocheng, Shandong Province. To confirm the presence of phages, the samples were assessed using the dual-layered plate method. The findings indicated that only one of the soil samples harbored *P. syringae* phage, and the lysis area was the same (Figure 1A). To examine the biological characteristics of LDT325, the bacteriophage was isolated using the double-layer agar plate method. The findings revealed plaques with varying dimensions and types on the double-layer agar plate (Figure 1B). After six rounds of refinement, LDT325 displayed plaques with consistent dimensions and types on double-layer agar plates (Figure 1C). Thus, a phage that lysed *P. syringae* was isolated and refined.

### 3.2 Microscopic analysis and classification of the bacteriophage LDT325

**Figure 2:**
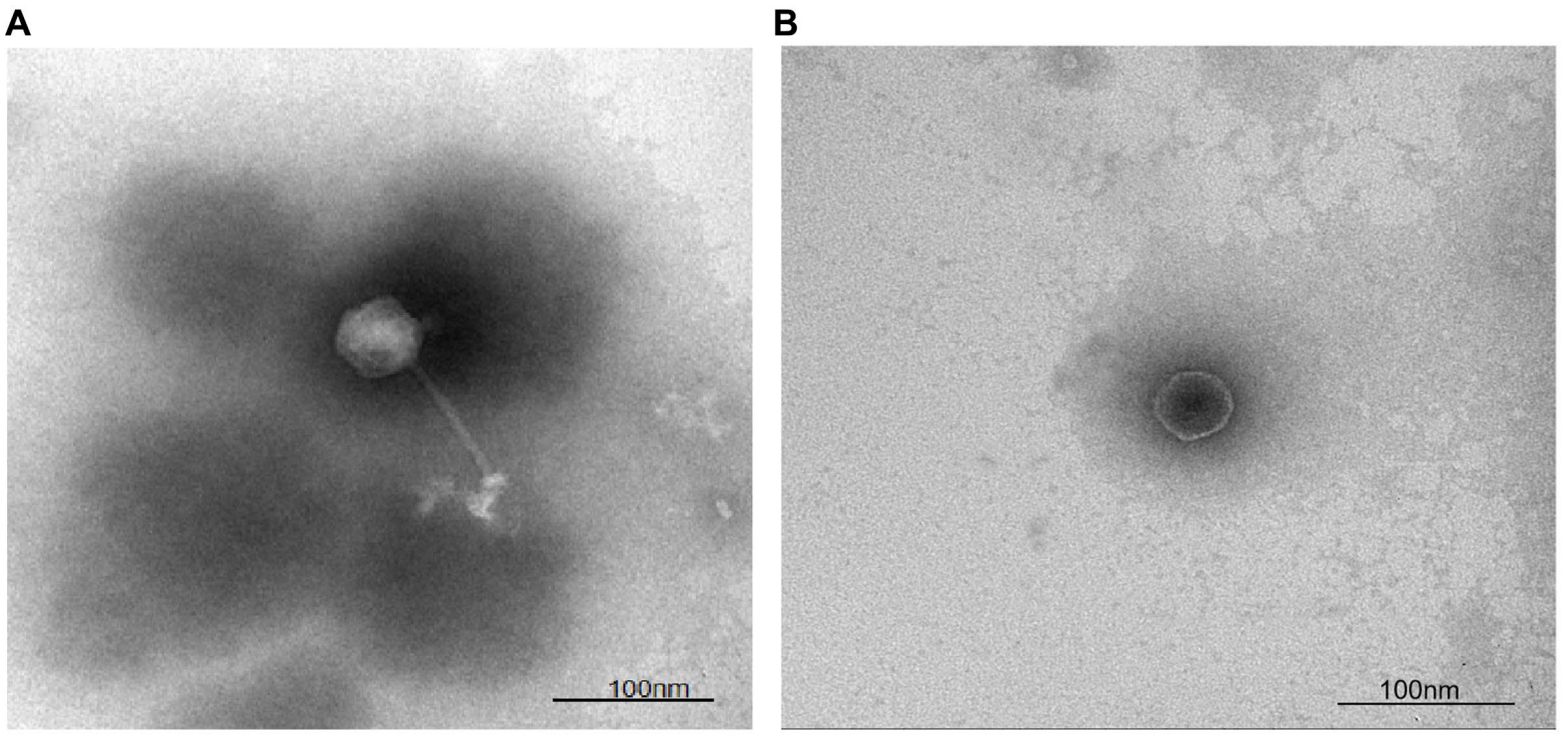
Micrograph and categorization of the phage vB_PsS_LDT325. **(A,B)** The transmission electron micrograph of the phage vB_PsS_LDT325 virus particles was negatively stained with 2% uranyl acetate. The scale bar represents 100 nm.

To categorize bacteriophage LDT325 into groups specific to morphotypes, its structure was examined using electron microscopy. Initially, LDT325 lysate was prepared via plate amplification, yielding a concentration of 1.45 × 10^10^ PFU/mL. The structure of bacteriophage LDT325 is shown in Figures 2A,B. Structurally, bacteriophage LDT325 contains an icosahedral head with a diameter of 53 ± 1 nm and elongated, nonretractable tails measuring 110 ± 1 nm. Based on morphological characteristics, bacteriophage LDT325 belongs to the family Siphoviridae and order Caudovirales, according to the recommendations of the International Committee on Taxonomy of Viruses (Fokine and Rossmann, 2014).

### 3.3 Biological properties of bacteriophage LDT325

**Figure 3:**
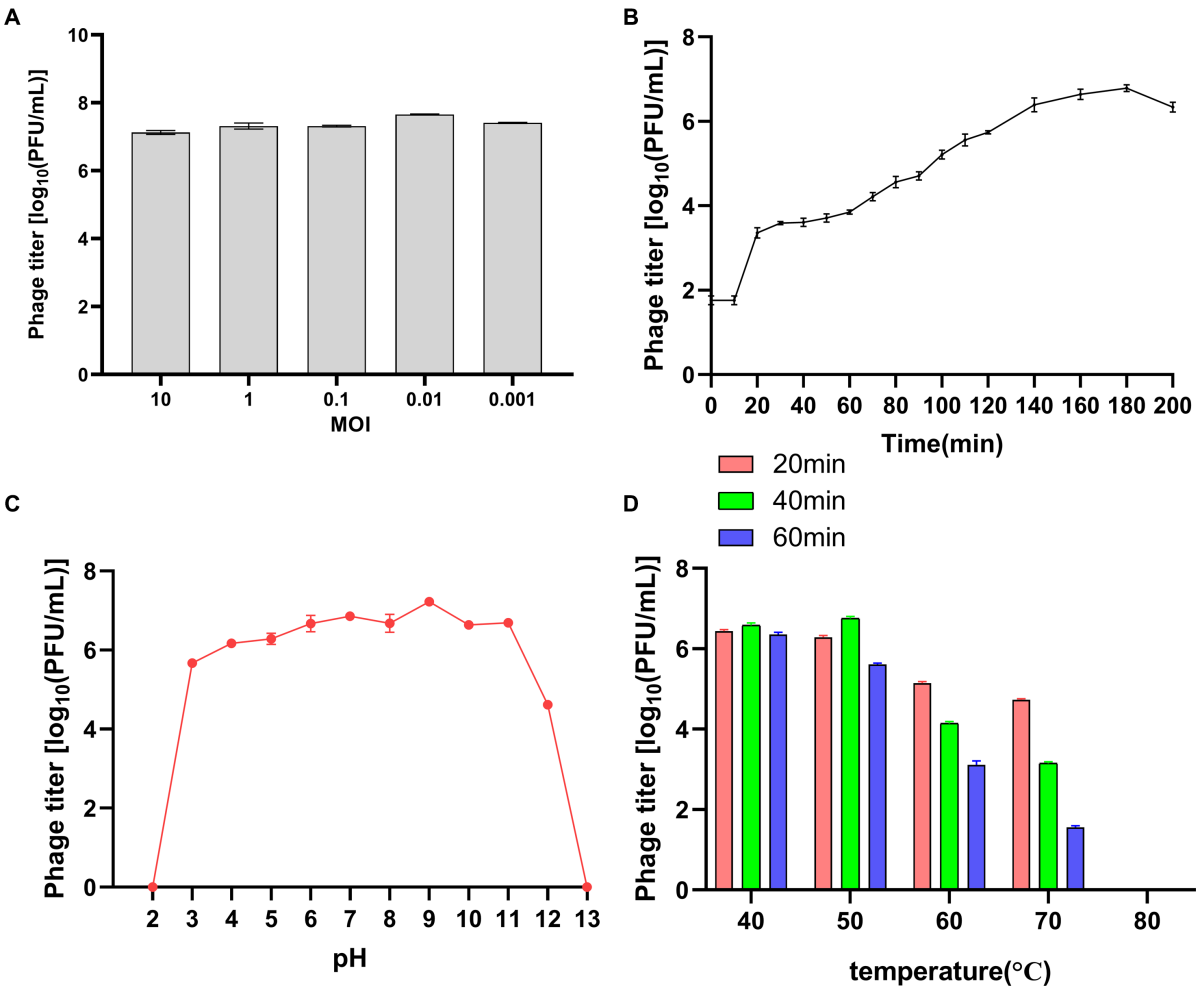
Biological characteristics of the phage vB_PsS_LDT325. **(A)** MOI of the phage vB_PsS_LDT325. Phage titers were measured at different MOIs. **(B)** One-step growth curve of the phage vB_PsS_LDT325. **(C)** The pH stability of the phage vB_PsS_LDT325. The phages were incubated in different acid–base environments for 1 h. **(D)** The thermal stability of the phage vB_PsS_LDT325. The phages were cultured in a water bath at different temperatures. **(A–D)** The phage titers were determined using the double-layer agar method and each data point represented the mean values ± standard deviations (SD) from at least three replicate experiments.

To determine the optimal MOI for bacteriophage LDT325, varying quantities of the phage were used to infect *P. syringae*. After incubation for 3 h, the phage titer was assessed. At an MOI of 0.01, the peak titer of phage LDT325 was 4.6 × 10^7^ PFU/mL (Figure 3A). Growth curve analysis of LDT325 indicated minimal alteration in the initial incubation period during the first 10 min, followed by a rapid growth period between 10 and 160 min, finally reaching a plateau after 160 min (Figure 3B). The latent period and burst size of the phage were 10 min and 17 PFU/cell, respectively. The stability of phage LDT325 in various environments was assessed based on its pH, thermal stability, and viability rate, which was determined by counting PFUs. In addition, pH significantly influenced adsorption, contagiousness, intracellular multiplication, and enhancement of the bacteriophage. Unfavorable pH values can disrupt lysozyme or other phage capsid proteins, ultimately hindering phage attachment to receptor sites on the host cell (Liu et al., 2021). Bacteriophage LDT325 was highly active at pH 3–11 but could not be detected at pH 1–2 and pH 13. Thus, phage LDT325 showed increased stability under alkaline conditions but displayed susceptibility to acidic or strong basic environments (Figure 3C). LDT325 maintained significant levels of infectivity after incubation in water at varying temperatures (Figure 3D). Phage stability declined gradually once the temperature exceeded 60°C, and phage activity steadily diminished with prolonged incubation under identical conditions. The phage was undetectable after incubation at 80°C for 1 h.

### 3.4 Impact of chloroform and UV radiation on phage survival

**Figure 4:**
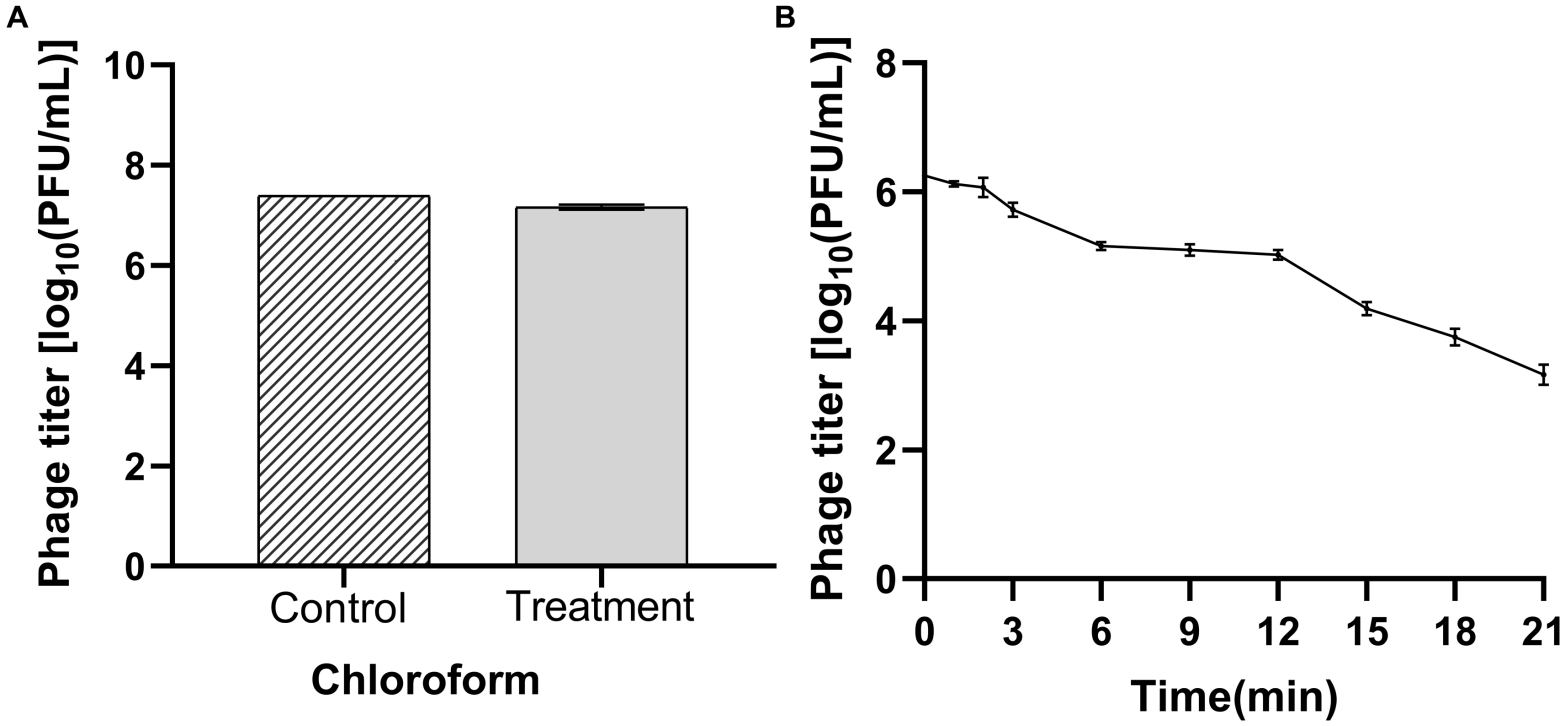
Determination of chloroform and ultraviolet sensitivity of phage vB_PsS_LDT325 **(A)** Chloroform stability: Phage vB_PsS_LDT325 was treated with chloroform (20%, vol/vol) for 12 h; **(B)** Ultraviolet light stability: Phage vB_PsS_LDT325 was exposed to UV light for 0, 1, 2, 3, 6, 9, 12, 15, 18 and 21 min. The data are represented as a mean of three replicates ± SD.

Chloroform treatment decreased the bacteriophage titer from 2.68 × 10^7^ to 1.55 × 10^7^ PFU/mL, with no significant difference. The bacteriophages were tolerant to chloroform (Figure 4A). Ultraviolet (UV) radiation kills viruses, mainly because ultraviolet light can directly destroy the nucleic acid molecules of viruses, causing DNA and RNA to break and damage, thereby preventing virus replication and transmission. The bacteriophage titer decreased from 1.78 × 10^7^ to 1.56 × 10^2^ PFU/mL with increased exposure time to UV (Figure 4B). Thus, bacteriophage LDT325 was sensitive to UV light.

### 3.5 Analysis of bacteriolytic activity *in vitro*

**Figure 5:**
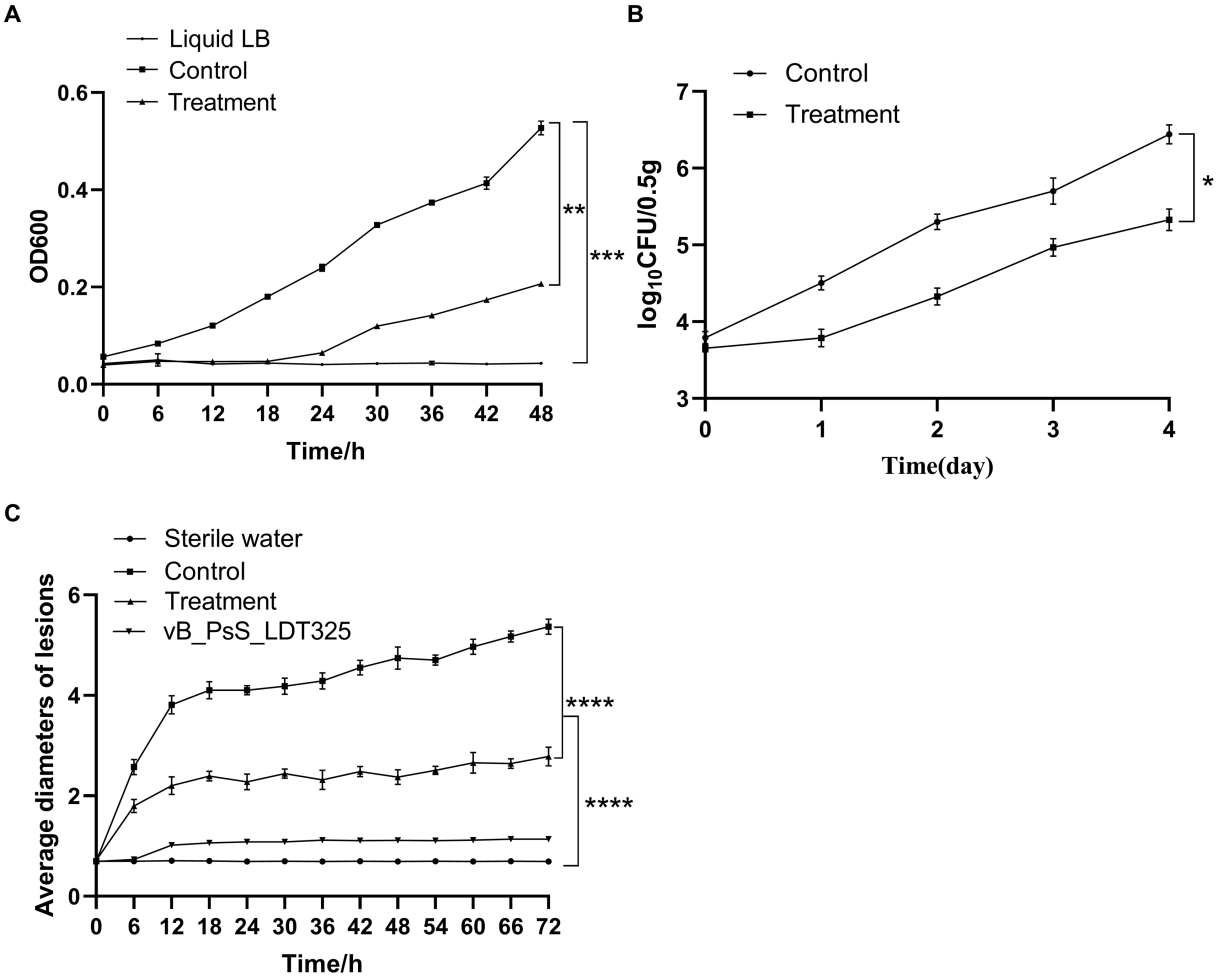
The biological control experiment of bud blight disease **(A)** Bacteriostasis experiment of phage *in vitro*. Asterisks signs indicate a statistically significant difference (*p* < 0.05), in which “*” (**p < 0.01) is compared with the phage-treated group, (*** *p* < 0.001) compared with the LB liquid medium group). **(B)** Bacteriostasis experiment of phage *in vivo*. Asterisks signs indicate a statistically significant difference (*p* < 0.05), in which “*” (*p < 0.05), is compared with the phage-treated group. **(C)** Change of lesion length on the leaves of living tea plant after inoculation with phage. Asterisks signs indicate a statistically significant difference (*p* < 0.05), in which “*” (**** *p* < 0.0001), is compared with the phage-treated and sterile water group. The data are represented as a mean of three replicates ± SD.

The potential application of bacteriophage LDT325 in inhibiting *P. syringae* was evaluated. To assess the duration of LDT325 bactericidal activity against *P. syringae*, cellular density (OD600) was monitored for 48 h (Figure 5A). The concentration of *P. syringae* devoid of bacteriophage increased by 0.34 after 48 h. After 48 h phage therapy, the bacterial load was markedly reduced in treated cultures compared to untreated cultures.

### 3.6 Analysis of phage treatment experiments *in vivo*

The *in vivo* biological control test results are shown in Figure 5B. The control and experimental groups exhibited notable differences in bacterial count at the same time point. After 4 days of treatment, bacterial CFU was 10^6^ in tea leaves untreated with phage. However, in tea leaves treated with phages, the bacterial CFU was 10^5^. Thus, LDT325 was effective against *P. syringae*.

### 3.7 Determination and analysis of lesion length of tea leaves

Treatment effect analysis of the *P. syringae* phage was initially performed on the leaves of Longjing No. 43. Under needling conditions, tea leaves were inoculated with sterile water and the bacteriophage did not yield any symptoms on the tea leaves. However, the trend of lesion length on tea leaves inoculated with *P. syringae* significantly differed between the experimental groups. The wounds were brown, and their sizes exhibited substantial variability among the four groups. After 72 h inoculation, wound width in the untreated group was 5.2 to 5.4 mm. The wound width in the experimental group ranged from 2.7 to 2.9 mm, suggesting that LDT325 is highly effective against *P. syringae* (Figure 5C).

### 3.8 Pathological section analysis

**Figure 6:**
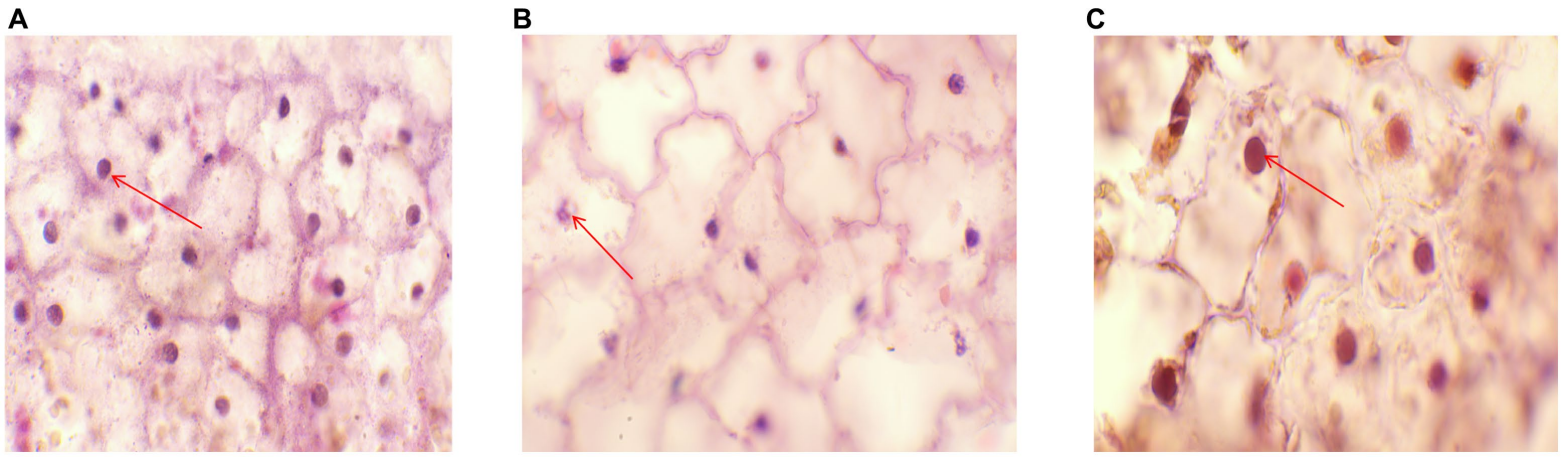
Pathological section **(A)** Histopathological images of plants in sterile water group. The red arrow indicates the nuclear region (HE,100×). **(B)** Histopathological images of the control group. The red arrow indicates the nuclear lesion area (HE,100×). **(C)** Histopathological images of phage treatment group. The red arrow indicates the nuclear region (HE,100×).

Ultrastructural and micropathological analyses of pathological sections of the sterile water group revealed clear cell walls and nuclei, with the nuclei appearing round and blue. The cytoplasm was pale red in color. The nucleus was scattered throughout the tissue (Figure 6A). These characteristics are specific to the internal morphological structures of normal leaves. Pathological sections of the control group inoculated with *P. syringae* showed nuclear rupture, indicating nuclear necrosis (Figure 6B). In the experimental groups, cell wall boundaries were clearer, and nuclei appeared round and blue, without shrinkage. The cytoplasm was pale red in color. The nucleus was scattered throughout the tissue (Figure 6C), suggesting that LDT325 is highly effective against *P. syringae*.

## 4 Discussion

Eliminating *P. syringae* from tea plants is essential for the normal growth of plants. However, current treatment methods for bud blight remain scarce. Some studies have shown that phages can successfully control plant bacterial diseases (Monk et al., 2010; Fujiwara et al., 2011; Nguyen et al., 2012). However, there are only a few reports have evaluated the effectiveness of phage inhibition in *P. syringae* infection through *in vitro* and *in vivo* experiments and pathological section analysis.

Phages are ubiquitous in the environment and are particularly enriched in the soil, with numbers reaching as high as 10^10^/g of the dry weight (Williamson et al., 2017). Bacteriophages have been proposed for treating plant bacterial diseases since the early 20th century (Cisek et al., 2017). However, in the 1940s, with the emergence and use of antibiotics, phage therapy suffered setbacks (Chanishvili, 2012). Reliance on antibiotic-based control measures has resulted in a significant decrease in efficacy due to the natural development of antibiotic resistance in bacteria (Altimira et al., 2012), and the emergence of resistant bacterial strains has prompted the investigation of alternative methods for combating bacterial diseases. Phage therapy (Holtappels et al., 2021) has been rediscovered as one of the most promising strategies for plant pathogen biocontrol.

In this study, we used *P. syringae* as the host and isolated a bacteriophage, which was named LDT325, from a soil sample. The bacteriophage LDT325 structure was examined using an electronic microscope. Results revealed that the phage possessed a 20-sided head and elongated, noncontraction tails. As per the International Classification of Viruses, bacteriophage LDT325 belongs to the family Siphoviridae and order Caudovirales (Fokine and Rossmann, 2014).

Investigating the biological characteristics of phages is a prerequisite for their use in biological control. The biological properties of phage LDT325 were examined in this study. MOI is an intrinsic characteristic of phages, and different phages have distinct optimal MOI. Determining the optimal MOI of phages is important for constructing one-step growth curves. The MOI is a major indicator of the ability of phages to lyse bacteria and may be used in subsequent large-scale phage production. In this study, the MOI for the phage was 0.01. Consistently, for the phage SoKa, which also infects *P. syringae*, the optimal MOI was determined to be 0.01 (Pinheiro et al., 2020). The latent period and outbreak size of the isolated bacteriophage were 10 and 150 min, respectively. The latent period of phage LDT325 was shorter than that of phage PHB09, which is also specific to *P. syringae* (Liu et al., 2021). Therefore, phage LDT325 is more conducive to practical application. The heat and pH resistance of the phage LDT325 were determined for its application as a biological control. The phage was stable at 40°C to 60°C, and its titer decreased when the temperature exceeded 60°C. Endersen discovered that phages leB, leE, and leN (specific to *Cronobacter sakazakii*) experienced a loss of activity after exposure to 60°C for 1 h. In contrast, the phages demonstrated enhanced resistance to high temperatures (Yin et al., 2019). At pH 3 to 11, the titer of bacteriophage LDT325 was stable, and at pH 2, the phage was inactivated. Compared with phages RpT1 and RpY2, which have been proposed as biocontrol agents against *Ralstonia pseudosolanacearum* invasion in tomato plants, phage LDT325 has a better pH tolerance range, indicating that it is quite stable in soil and can remain infectious for a long time (Frampton et al., 2014). Previous studies have consistently reported that bacteriophages exhibit greater resistance to alkaline conditions than acidic ones (Zhang et al., 2018; Jiang et al., 2021). Bacteriophage LDT325 showed no significant titer change after incubation with chloroform for 1 h, indicating strong tolerance to this reagent, which is consistent with the results of previous studies (Nordeen et al., 1983). The detrimental impacts of UV radiation on microbes have been extensively recognized and explored in studies on microbial ecology (Paul et al., 1997). Long-term UV irradiation significantly affected the phage titer, consistent with previous studies (Gašić et al., 2018).

The effectiveness of bacteriophage LDT325 in eliminating *P. syringae* was assessed by investigating the dynamics of bacteriophage–host duplication. *In vitro*, 48 h of phage therapy markedly diminished the bacterial load compared with untreated cultures. In *in vitro* experiments, compared with phage PHB09, phage LDT325 showed a significant antibacterial effect (Liu et al., 2021). For the effective application of phages in controlling bacteria that cause plant diseases, the bacteriophage must interact with the host on the leaf surface (Vieira et al., 2012). Therefore, *in vivo* phage trials were conducted using artificially infected tea leaves. Phage LDT325 substantially reduced the levels of *P. syringae* compared with untreated bacterial controls. Consistent with previous studies, phages decreased the bacterial burden of *P. syringae* inside the leaf and reduced damage to the leaf tissue (Flores et al., 2020). Koizumi used the needle-prick method to demonstrate a significant therapeutic effect of the phage in inhibiting *P. syringae*. After 72 h, compared with the control group, the average lesion diameter was reduced by 50% on tea leaves in the experimental group. Based on *in vivo* and *in vitro* control effects, LDT325 maintained stable cleavage activity against Psa, highlighting the potential of LDT325 in the biological control of Psa infection. The lytic pattern of Psa phages (KHUφ34 and KHUφ38) is highly similar (Yu et al., 2016; Park et al., 2018). Pyknotic and fragmented nuclei were observed in cells undergoing necrosis. During the early stage of cell death, the cell membrane becomes permeable. As apoptosis progresses, the nucleus undergoes further condensation and eventually breaks apart within the intact cell membrane—a phenomenon known as karyorrhexis (Kar and Sivamani, 2015). The morphological method is one of the most intuitive and reliable methods for detecting nuclear rupture. After staining, electron microscopy was used to observe the tissue and determine the occurrence of nuclear rupture based on cell morphology or staining type. Nuclear rupture refers to the destructive fragmentation of the nucleus of dying cells. Chromatin was irregularly distributed in the cytoplasm. Nuclear dissolution occurs after nuclear rupture, which may result from cell death and necrosis. The ultrastructure of leaf tissue sections was observed by electron microscope. Pathological sections of the control group inoculated with *P. syringae* showed nuclear rupture, indicating nuclear necrosis. These results were consistent with the characteristics of cell death and necrosis reported in the literature (Nunes and Moretti, 2017). In the phage treatment experiment, the cell wall boundary was found to be clearer. The nucleus was round and there was no nuclear rupture. This demonstrated that the phage protected the tea leaf tissue to reduce the occurrence of lesions. In this study, phage LDT325 effectively reduced the levels of *P. syringae*, indicating the development of a sustainable and environmentally friendly alternative to traditional copper and antibiotic treatments. Furthermore, the isolated bacteriophages showed optimal tolerance to pH and temperature and exerted good antibacterial effects in practical applications.

In summary, a bacteriophage against *P. syringae* with practical potential was isolated in this study. Phage vB_PsS_LDT325 exhibited superior anti-*P. syringae* activities and relatively high thermal and acid tolerance, showing great potential for controlling *P. syringae* in a natural environment. Although further characteristics of the phage and a deeper analysis of its practical properties are warranted, this study provides a promising approach to combating tea infections caused by *P. syringae*.

## Data availability statement

The original contributions presented in the study are included in the article/supplementary material, further inquiries can be directed to the corresponding author.

## Author contributions

LL: Data curation, Investigation, Writing – original draft. BW: Writing – review & editing. AH: Investigation, Writing – review & editing. HZ: Software, Writing – review & editing. YL: Writing – review & editing. LW: Writing – review & editing.
